# Shape Effect of Nanosize Particles on Magnetohydrodynamic Nanofluid Flow and Heat Transfer over a Stretching Sheet with Entropy Generation

**DOI:** 10.3390/e22101171

**Published:** 2020-10-18

**Authors:** Umair Rashid, Dumitru Baleanu, Azhar Iqbal, Muhammd Abbas

**Affiliations:** 1 Department of Modern Mechanics, CAS Key Laboratory of Mechanical Behavior and Design of Materials, University of Science and Technology of China, Hefei 230026, China; umair2014@mail.ustc.edu.cn; 2 Department of Mathematics, Faculty of Arts and Sciences, Cankaya University, Ankara 06530, Turkey; dumitru@cankaya.edu.tr; 3 Department of Medical Research, China Medical University, Taichung 40402, Taiwan; 4 Institute of Space Sciences, 077125 Magurele-Bucharest, Romania; 5 Mathematics and Natural Sciences, Prince Mohammad Bin Fahd University, Al Khobar 31952, Saudi Arabia; 6 Department of Mathematics, University of Sargodha, Sargodha 40100, Pakistan

**Keywords:** nanofluid, nanoparticles, analytical solution, magnetic field, entropy generation

## Abstract

Magnetohydrodynamic nanofluid technologies are emerging in several areas including pharmacology, medicine and lubrication (smart tribology). The present study discusses the heat transfer and entropy generation of magnetohydrodynamic (MHD) Ag-water nanofluid flow over a stretching sheet with the effect of nanoparticles shape. Three different geometries of nanoparticles—sphere, blade and lamina—are considered. The problem is modeled in the form of momentum, energy and entropy equations. The homotopy analysis method (HAM) is used to find the analytical solution of momentum, energy and entropy equations. The variations of velocity profile, temperature profile, Nusselt number and entropy generation with the influences of physical parameters are discussed in graphical form. The results show that the performance of lamina-shaped nanoparticles is better in temperature distribution, heat transfer and enhancement of the entropy generation.

## 1. Introduction

The entropy generation is related to thermo-dynamic irreversibility; entropy generation is common in every category of heat transfer processes. The entropy generation technique is introduced by Bejan [1,2]. The quantity of irreversibility equal to that of entropy can be measured during a process. Entropy analysis is an influential means to find the efficiency of a system or process. Limited studies on the entropy generation of turbulent nanofluid flow have been analyzed in the microchannel, stretching sheet and channels; the entropy generation in the microchannel and channel is studied more closely although the studies described can apply to nanofluid, fluid turbulent, or laminar flow [3]. Some researchers have worked on entropy generation by considering different types of geometry. Ko and Cheng [4] numerically studied the entropy generation in a channel wavy wall. According to the results of Ko and Cheng, amplitude of the wavy wall is increasing function of the entropy generation. Mahian et al. [5] presented the entropy generation of water-TiO_2_ and ethylene glycol-Al_2_O_3_ nanofluids between two circular cylinders. The obtained results displayed that the entropy generation has inverse relation with the volume fraction of nanofluid. Tshehla and Makinde [6] presented the entropy generation of steady flow between two concentric pipes. They demonstrated that entropy generation is directly relational to Brinkman number. Govindaraju et al. [7] analyzed the impact of slip magnetohydrodynamic nanofluid on entropy generation in a stretching sheet. The authors found that the entropy generation reduces in the flow system in the presence of slip parameter and volume fraction. Govindaraju et al. [1] presented the entropy generation study of an incompressible magnetohydrodynamic of viscous nanofluid flow over a stretching sheet by considering various categories of nanoparticles in the water-based fluid. They concluded that entropy generation has a direct relation with Hartmann number, magnetic parameter and dimensionless group parameter. Berrehal and Maougal [8] presented entropy generation analysis for nanofluid flow over a wedge with convective boundary condition and thermal radiation. The authors found that the entropy generation can be decreased with deducing convection through boundary and with increase radiation parameter. Malvandi et al. [9] discussed analytical solution of entropy generation for nanofluids over a flat plate. They demonstrated that the entropy generation depends on Prandtl number, Reynold number, Eckert number and volume fraction. Acharya et al. [10] presented the entropy generation and heat transfer in a regenerative cooling channel of a rocket engine. Govindaraju et al. [2] studied analytically the entropy generation of nanofluid flow over a stretching sheet with a uniform heat source-sink and inclined magnetic field. The results exposed that entropy generation has a direct relation with Ec and ϕ, while entropy generation decreases with the increase in A.

Magnetohydrodynamics attracted the attention of researchers due to natural phenomena, i.e., geophysics, astrophysics to several applications in engineering such as electromagnetic casting, liquid metal, plasma confinement and so on [11]. Nadeem et al. [12] discussed two-dimensional boundary layer flow over a stretching sheet with the effect of magnetohydrodynamics. The results exposed that when increasing the value of the Prandtl number and magnetic parameters, an opposite behavior is seen in the Sherwood number and Nusselt number. Rudraiah et al. [13] numerically examined the impact of magnetic field on natural convection. The authors noted that heat transfer rate decreases with the effect of the magnetic field. Abbas et al. [14] numerically discussed the effects of magnetohydrodynamics over a stretching continuous sheet in a rotating fluid.

The concept of a nanofluid was introduced by Choi [15] who examined notable results with several possibilities of usage. Nanofluids are the latest class of nanotechnology concerning nanoparticles dispersed in base fluid. The heat transfer fluid became a significant interest in research due to various applications. The heat transfer rate of a nanofluid is greater due to greater thermal conductivities as compared to the base fluids [16]. There are several papers on water-Ag nanofluid flow by many researchers. Atashafrooz [17] studied the impact of water-Ag nanofluid flow over an inclined step by using numerical technique. Upreti et al. [18] examined the water-Ag nanofluid over a flat porous plate with influences of injection/suction, heat absorption/generation and viscous-ohmic dissipation. Suleman et al. [19] discussed the water-Ag nanofluid flow in a stretching cylinder with the effects of homogenous-heterogeneous reaction and Newtonian heating.

The geometry effect of nanoparticles is very important to change the thermal conductivity of the nanofluid [20]. Several researchers have worked on Ag-water nanofluid flow. To the best of our knowledge, there is no such research on the shape effect of nanoparticles on magnetohydrodynamic Ag-water nanofluid flow over a stretching sheet with entropy generation. The motivation of the present paper is to investigate the shape effect of Ag nanoparticles on magnetohydrodynamic Ag-water nanofluid flow and heat transfer with entropy generation. Finally, graphs of velocity, temperature, Nusselt number and entropy generation are plotted and all their aspects are discussed.

This study is organized as follows: in the first section, we construct the mathematical formulation for the proposed model. The numerical solution of proposed model is obtained by homotopy analysis method in Section 3. The entropy generation is discussed in Section 4. The computational results are reported in Section 5. Finally, conclusions are described in Section 6.

## 2. Mathematical Formulation

This section considers entropy analysis for a two-dimensional steady nanofluid laminar flow over a linear semi-infinite stretching sheet. Furthermore, it is assumed that $B_{0}$ magnetic field is imposed normally to the stretching sheet. It is also assumed that the nanofluid is water based containing various shapes of Ag nanoparticles. The thermo-physical properties of Ag nanoparticles and water are presented in Table 1. The values of nanoparticles shape related parameter is presented in Table 2. The governing equations are

(1) $$\frac{\partial \left(u\right)}{\partial x}\;+\;\frac{\partial \left(v\right)}{\partial y}\;=\;0,$$

(2) $$u\frac{\partial u}{\partial x}\;+\;v\frac{\partial u}{\partial y}\;=\;\frac{\mu_{n_{f\;}}}{\rho_{n_{f}}}\frac{\partial^{2}u}{\partial y^{2}}\;+\;\frac{\sigma_{n_{f}}B_{0}{}^{2}u^{2}}{\rho_{n_{f}}},$$

(3) $$u\frac{\partial T}{\partial x}\;+\;v\frac{\partial T}{\partial y}\;=\;\frac{k_{n_{f}}}{{\left(\rho Cp\right)}_{nf}}\frac{\partial^{2}T}{\partial y^{2}}.$$

The boundary value conditions are

(4) $$u\;=\;U_{w}\left(x\right)\;=\;ax,v=0,T\;=T_{w},\;\mathrm{at}\;y=0,\;\;u\;=\;v\;=\;0,\;T\;=\;T_{\infty },\;\mathrm{at}\;y\;\to \infty .$$

The nondimensionalize variables are defined as

(5) $$x\;=\;\frac{x}{\sqrt{\frac{\nu_{f}}{a}}},\;\;y\;=\;\frac{y}{\sqrt{\frac{\nu_{f}}{a}}},\;U\;=\;\frac{u}{\sqrt{a\nu_{f}}},\;\;V\;=\;\frac{v}{\sqrt{a\nu_{f}}},\;\;\theta \left(\eta \right)\;=\;\frac{T-T_{\infty }}{T_{f}-T_{\infty }}$$

After substituting Equation (5) into Equations (1)–(3) we obtain

(6) $$f^{\prime \prime \prime }+B1\left\{B2\left(ff^{\prime \prime }-f^{\prime \prime 2}\right)-Mf^{\prime }\right\}\;=\;0,$$

(7) $$\theta^{\prime \prime }+Pr\frac{B3}{B4}f\theta^{\prime \;}\;=\;0.$$

The corresponding boundary value conditions are

(8) $$f\left(0\right)\;=\;f^{\prime }\left(0\right)\;=\;1,\;f^{\prime }\left(\infty \right)\;=\;0,\;\theta \left(0\right)\;=\;1,\theta \left(\infty \right)\;=\;0,$$

where,

$$B1={\left(1-\phi \right)}^{2\cdot 5},\;B2=\left(1-\phi \right)+\frac{\rho_{s}}{\rho_{f}}\phi ,\;B3=\left(1-\phi \right)+\frac{{\left(\rho Cp\right)}_{s}}{{\left(\rho Cp\right)}_{f}}\phi ,\;B4=\frac{k_{n_{f}}}{k_{f}}=\frac{\left[k_{s}+\left(m-1\right)k_{f}\right]-\left(m-1\right)\phi \left(k_{f}-k_{s}\right)}{\left[k_{S}+\left(m-1\right)k_{f}\right]+\phi \left(k_{f}-k_{s}\right)}.$$

The important physical quantity of interest, the Nusselt number (*N_u_*) is defined as [1]

(9) $$N_{u_{x}},\;=\;\frac{x\;q_{w}\left(x\right)}{k_{f}\left[\;T_{f}-\;T_{\infty }\right]},$$

where $\;q_{w}\left(x\right)$ is given by

(10) $$\;q_{w}\left(x\right)\;=\;-k_{n_{f}}{\left(\frac{\partial T}{\partial y}\right)}_{y\;=\;0}.$$

Substituting Equation (9) into Equation (10), we obtain

(11) $$\frac{N_{u_{x}}}{\sqrt{Re_{x}}}\;=\;-\frac{k_{n_{f}}}{k_{f}}\theta^{\prime }\left(0\right).$$

## 3. Solution via Homotopy Analysis Method

For the solution of the homotopy analysis method, we choose the following auxiliary linear operators.

(12) $$L_{f}=f^{\prime \prime \prime }-f^{\prime },\;\;\;\;\;\;L_{\theta }=f^{\prime \prime }-f,$$

which satisfied the following properties

(13) $$L_{f}\left[Z_{1}+Z_{2}e^{\eta }+Z_{3}e^{-\eta }\right]=0,\;\;\;\;\;L_{\theta }\left[Z_{4}e^{\eta }+Z_{5}e^{-\eta }\right]=0,$$

where $Z_{1}\;$, $Z_{2}\;$, $Z_{3}\;$, $Z_{4}$ and $Z_{5}$ are arbitrary constants.

It is assumed that *p* ∈ [0, 1] denotes an embedding parameter and ℏ *f,* ℏ *θ ≠* 0 are convergence control parameters.

The zeroth order deformations are

(14) $$\left(1-p\right)L_{f}\left[\hat{f}\left(\eta ,p\right)-f_{0}\left(\eta \right)\right]=p\hbar_{f}N_{f}\left[\hat{f}\left(\eta ,p\right),{\hat{\theta }}_{0}\left(\eta ,p\right)\right],$$

(15) $$\hat{f}\left(0,p\right)=0,\;{\hat{f}}^{\prime }\left(0,p\right)=1,\;\hat{f}\left(\infty ,p\right)=0.\;\left(1-p\right)L_{\theta }\left[\hat{\theta \;}\left(\eta ,p\right)-\theta_{0}\left(\eta \right)\right]=p\hbar_{\theta }N_{\theta }\left[\;{\hat{\theta }}_{0}\left(\eta ,p\right),\hat{f}\left(\eta ,p\right)\right],\;\hat{\theta }\left(0,p\right)=1,\;\hat{\theta }\left(\infty ,p\right)=0,$$

and

(16) $$\begin{array}{ll} N_{f}\left[\;\hat{f}\left(\eta ,p\right),\hat{\theta }\left(\eta ,p\right)\right] & \\ & =\frac{\partial^{3}\;\hat{f}\left(\eta ,p\right)}{\partial \eta^{3}}+B1\{B2\left(\hat{f}\left(\eta ,p\right)\frac{\partial^{2}\;\hat{f}\left(\eta ,p\right)}{\partial \eta^{2}}-\frac{\partial^{2}\;\hat{f}\left(\eta ,p\right)}{\partial \eta^{2}}\frac{\partial^{2}\;\hat{f}\left(\eta ,p\right)}{\partial \eta^{2}}\right)\\ & -M\frac{\partial \hat{f}\left(\eta ,p\right)}{\partial \eta }\}=0, \end{array}$$

(17) $$N_{\theta }\left[\;\hat{\theta }\left(\eta ,p\right),\;\hat{\;f}\left(\eta ,p\right)\right]=\;\frac{\partial^{2}\hat{\theta }\left(\eta ,p\right)}{\partial \eta^{2}}+Pr\frac{B3}{B4}\hat{f}\left(\eta ,p\right)\frac{\partial \hat{\theta }\left(\eta ,p\right)}{\partial \eta }=0.$$

Due to Taylor’s series with respect to *p*, we have

(18) $$f\left(\eta ,p\right)=f_{0}\left(\eta \right)+\overset{\infty }{\underset{m=1}{\sum }}f_{m}(\eta )p^{m},\;\;\;\;\theta \left(\eta ,p\right)=\theta_{0}\left(\eta \right)+\overset{\infty }{\underset{m=1}{\sum }}\theta_{m}(\eta )p^{m},$$

where,

(19) $$\;f_{m}\left(\eta \right)={\left.\frac{1}{m!}\frac{\partial^{m}f\left(\eta ,p\right)}{\partial \eta^{m}}\right|}_{p=0},\;\;\theta {\;}_{m}\left(\eta \right)={\left.\frac{1}{m!}\frac{\partial^{m}\;\theta \;\left(\eta ,p\right)}{\partial \eta^{m}}\right|}_{p=0},$$

and thus, higher order deformation problems are

(20) $$L_{f}\;\left[f_{m}\left(\eta \right)-\chi_{m}f_{m-1}\left(\eta \right)\right]=\hbar_{f}R_{f}^{m}\left(f_{m-1}\left(\eta \right),\theta_{m-1}\left(\eta \right)\right),\;f_{m}\left(0\right)=0,f_{m}^{\prime }\left(0\right)=0,f_{m}^{\prime }\left(\infty \right)=0,$$

(21) $$LL_{f}\;\left[\theta_{m}\left(\eta \right)-\chi_{m}\theta_{m-1}\left(\eta \right)\right]=\hbar_{\theta }R_{\theta }^{m}\left(\theta_{m-1}\left(\eta \right),f_{m-1}\left(\eta \right)\right),\;\theta_{m}\left(0\right)=0,\theta_{m}\left(\infty \right)=0.$$

Here,

(22) $$\chi_{m}=\left\{\begin{array}{c} 0\;\;\;\;\;\;\;m\;\leq \;1\;\\ 1\;\;\;\;\;\;\;\;\;\;m>1 \end{array}\;\right.$$

and

(23) $$R_{f}^{m}f_{m}\left(\eta \right)=f_{m-1}^{\prime \prime \prime \prime }\left(\eta \right)+B1\{B2\overset{m-1}{\underset{z=0}{\sum }}f_{z}f_{m-1-z}^{\prime \prime }-\frac{1}{2}\overset{m-1}{\underset{z=0}{\sum }}f_{z}^{\prime }f_{m-1-z}^{\prime })+f_{z}f_{m-1}^{\prime }\left(\eta \right)\},$$

(24) $$R_{f}^{m}\theta_{m}\left(\eta \right)=\theta_{m-1}^{\prime \prime }+Pr\frac{B3}{B4}\overset{m-1}{\underset{z=0}{\sum }}f_{z}\theta_{m-1-z}^{\prime }.$$

## 4. Entropy Generation

The entropy generation in the influence of $B_{0}$ magnetic field is defined as:

(25) $$\;S_{a}=\frac{k_{n_{f}}}{\;T_{\infty }}\left[{\left(\frac{\partial T}{\partial x}\right)}^{2}+{\left(\frac{\partial T}{\partial y}\right)}^{2}\right]+\frac{\mu_{n_{f\;}}}{\;T_{\infty }}{\left(\frac{\partial u}{\partial y}\right)}^{2}+\frac{\sigma_{n_{f}}B_{0}{\;}^{2}u^{2}}{\;T_{\infty }}.$$

The first term of Equation (25) expresses the entropy generation due to heat transfer, the middle term expresses the viscous dissipation and the last term denotes the entropy generation due to magnetic field effect. The entropy generation rate in dimensionless method is described as the ratio of volumetric entropy ($S_{a}$) to characteristic entropy generation rate ($S_{b}$), the characteristic entropy generation rate is defined as:

(26) $$\;S_{b}=\frac{k_{n_{f}}{\left(\nabla T\right)}^{2}}{x^{2}\;T_{\infty }^{2}}.$$

The entropy number is defined as [1]

(27) $$N=\frac{\;S_{a}}{\;S_{b}},$$

(28) $$N=\theta^{\prime 2}\left(\eta \right)+\frac{B}{\Omega }f^{\prime \prime 2}\left(\eta \right)+\frac{B\;{\left(\;H_{a}\right)}^{2}}{\;\Omega R_{e}}f^{\prime 2}\left(\eta \right).$$

## 5. Results and Discussion

**Figure 1:**
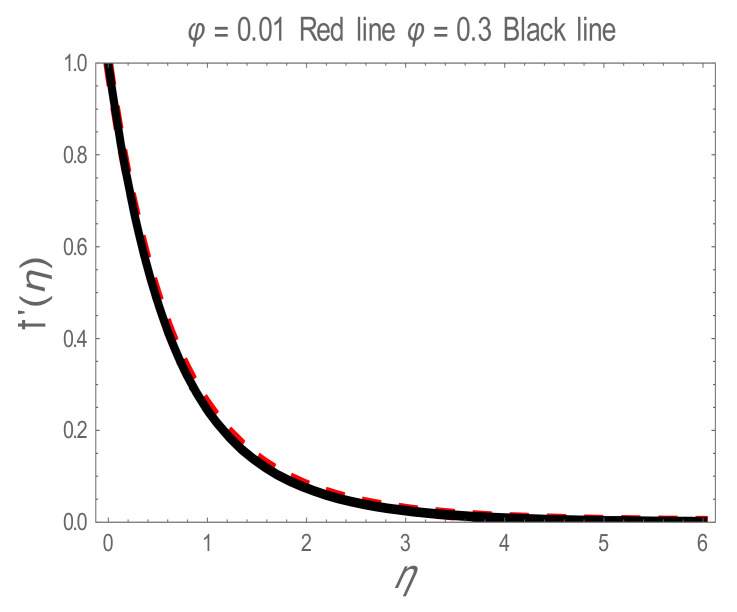
$f^{\prime }\left(\eta \right)$ for values of solid volume fraction (ϕ) .

**Figure 2:**
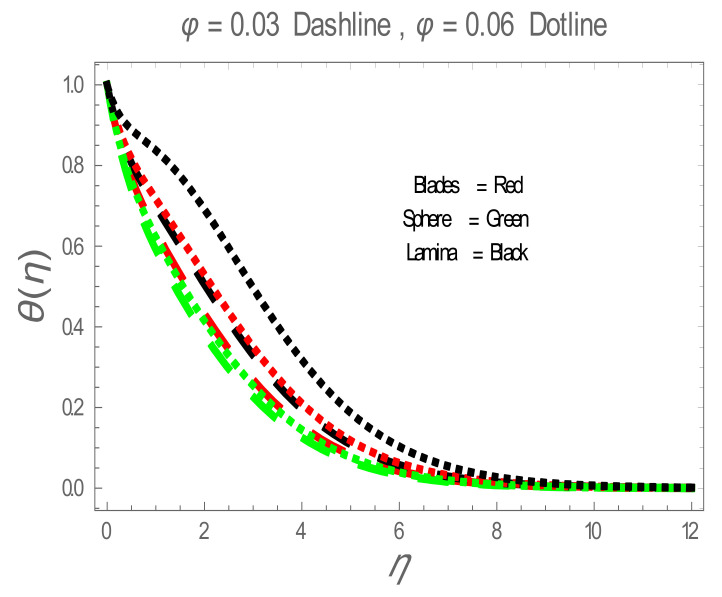
$\theta \left(\eta \right)$ for values of ϕ .

**Figure 3:**
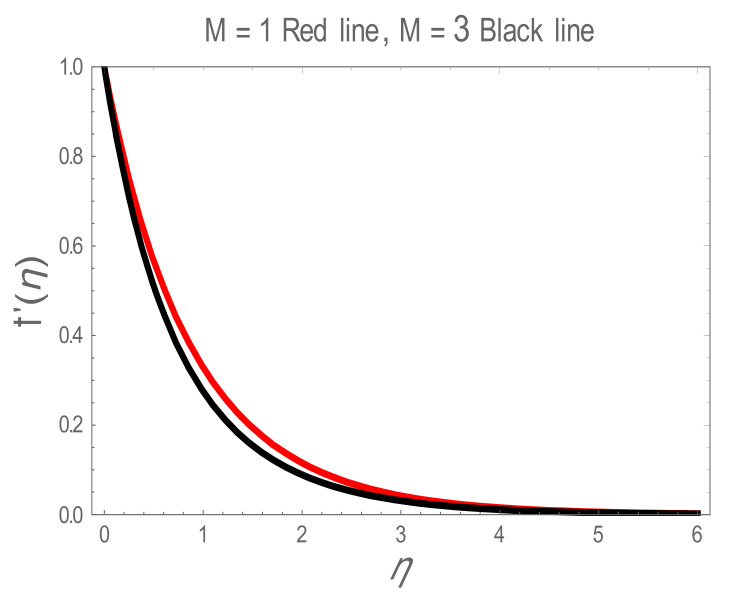
$f^{\prime }\left(\eta \right)$ for values of magnetic parameter (M).

**Figure 4:**
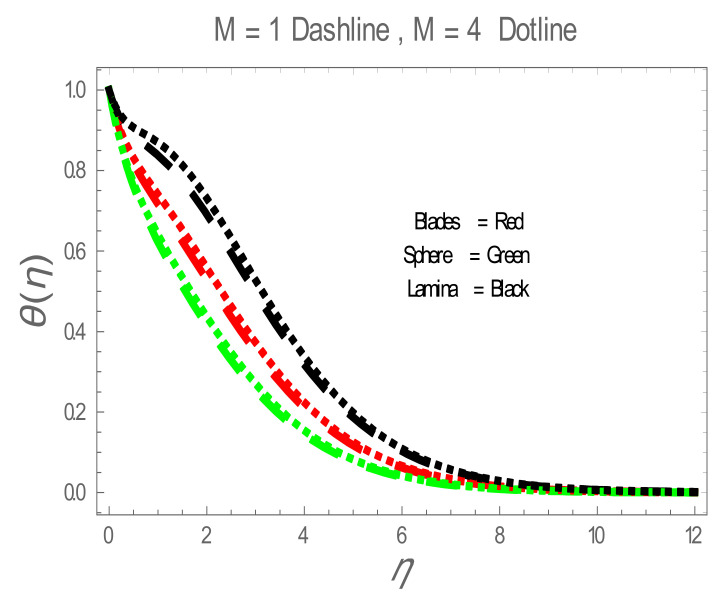
$\theta \left(\eta \right)$ for values of M.

**Figure 5:**
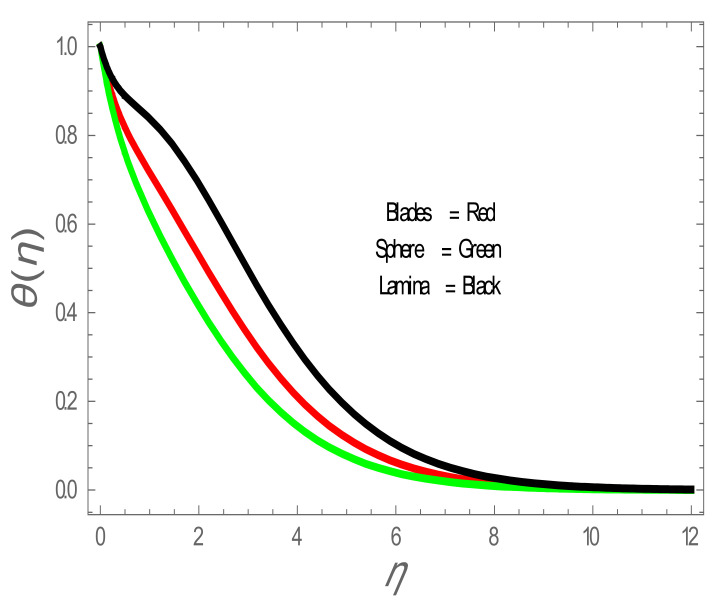
$\theta \left(\eta \right)$ with effect of nanoparticles shape.

**Figure 6:**
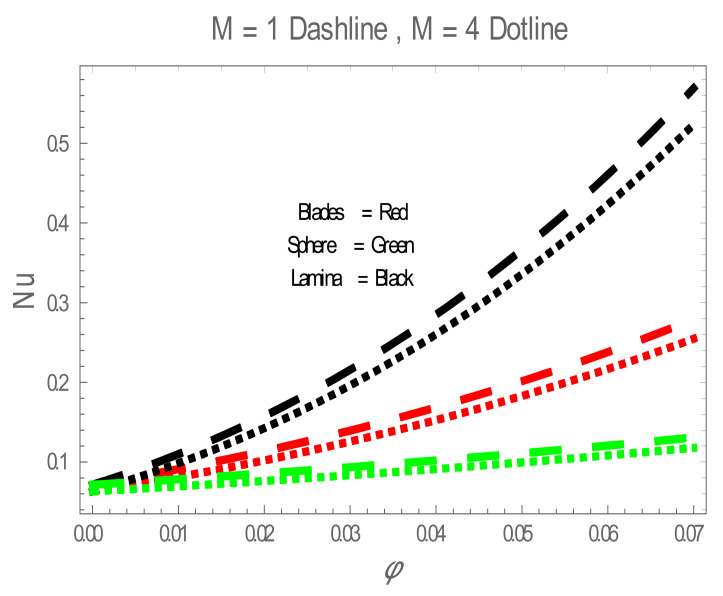
Nu for values of M.

**Figure 7:**
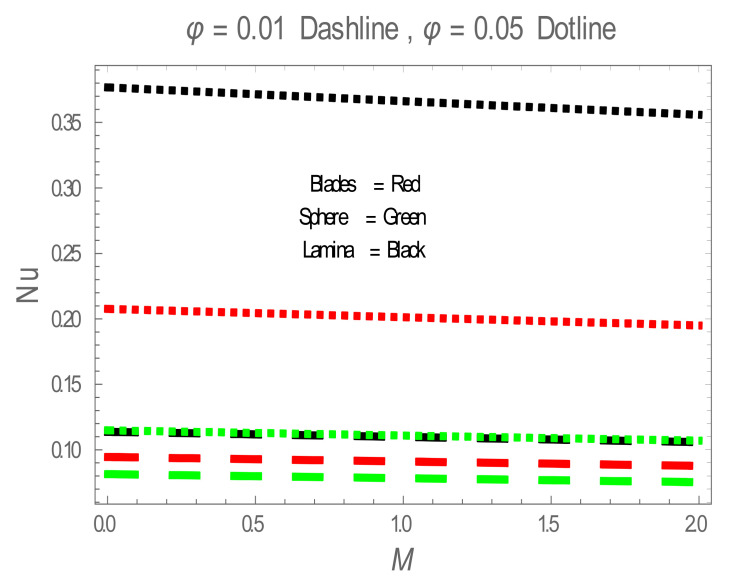
Nusselt number (Nu) for values of ϕ . The impact of nanoparticles shapes in entropy generation profile is displayed in Figure 8. Figure 8 illustrates that lamina-shaped nanoparticles have a better performance on the entropy profile. The impact of M on entropy number is displayed in Figure 9. It is observed that near the wall, the entropy number intensifies with the increase in the M. It is due to the Lorentz force which is produced due to M opposing the motion of the nanofluid; also Lorentz force heating temperature equation heat source to the boundary layer of the nanofluid. The influence of M produces the entropy in nanofluid. The variation in entropy number with the influences of $\;B\Omega^{-1}\;$is presented in Figure 10. It is observed that the adjacent entropy number near the wall is attributed to a fraction of the nanofluid increasing with an increase in entropy numbers. Figure 11 represents the entropy number with the variation of $\;R_{e}\;$. Figure 11 displays that near the wall, entropy number intensifies with an increase in the $\;R_{e}$ resulting in an increase in the nanofluid fraction. Figure 12 demonstrates the variation of entropy numbers with the influences of ϕ . It is observed that ϕ has a direct relationship with entropy number, since the lower energy of dissipation results from the gradient of the gradual velocity near the wall.

In the present section, the effects of several parameters, such as magnetic parameter (M) and solid volume fraction (ϕ), on velocity $f^{\prime }\left(\eta \right),\;$temperature $\theta \left(\eta \right)$ and Nusselt number (Nu) are displayed in Figure 1, Figure 2, Figure 3, Figure 4, Figure 5, Figure 6 and Figure 7. The effect of nanoparticles has also been plotted. The variation of velocity and temperature profiles with the influence of ϕ is displayed in Figure 1 and Figure 2. In Figure 1 and Figure 2, a decrement in velocity and temperature of nanofluid increase with an increase in the ϕ can be seen. It is noticed that thermal conductivity of nanofluid has direct relation with solid volume fraction, thus the thermal boundary layer thickness is increased. It is also noted from Figure 2, performance of lamina-shaped nanoparticles on temperature distribution is better than blade and sphere shapes nanoparticles. The velocity and temperature profiles with influences (M) are depicted in Figure 3 and Figure 4. It is observed that velocity and temperature of the nanofluid have an inverse and direct relationship with M, respectively. Physically, the Lorentz force produced due to M opposes the motion of the nanofluid, also Lorentz force heating temperature equation will provide an extra heat source to the boundary layer of the nanofluid. It is observed from Figure 4 that the performance of lamina-shaped nanoparticles on temperature distribution is better than blade- and sphere-shaped nanoparticles. Figure 5 displays the effect of nanoparticles in temperature profile. Figure 5 shows that the performance of lamina-shaped nanoparticles on temperature distribution is better than blade- and sphere-shaped nanoparticles. This is because the nanoparticles in the form of a lamina have low viscosity and thermal conductivity compared to nanoparticles in the form of a blade and a sphere. The impacts of magnetic parameter (M) and solid volume fraction (ϕ) on Nusselt number (Nu) are plotted in Figure 6 and Figure 7. From Figure 6 and Figure 7, it is noted that lamina-shaped nanoparticles have a higher rate of heat transfer as compared to blade- and sphere-shaped nanoparticles.

Table 3 shows that excellent agreement has been found in the comparison of results with Govindaraju et al. [1] and Wang Results [21].

## 6. Conclusions

This paper explores the heat transfer and entropy generation of magnetohydrodynamic (MHD) Ag-water nanofluid flow over a stretching sheet with the effect of nanoparticles shapes. The homotopy analysis method is used for analytical solutions of velocity profile, temperature profile, Nusselt number and entropy generation. The fixed value of Prandtl number Pr = 6.2 is used. A agree ment has been obtained Lamina-shaped nanoparticles show dramatic role in the disturbance of temperature profile, heat transfer and increase in entropy generation, while the performance of sphere-shaped nanoparticle is lowest in disturbance of temperature profile, heat transfer and increase in entropy generation.

## Figures and Tables

**Figure 8:**
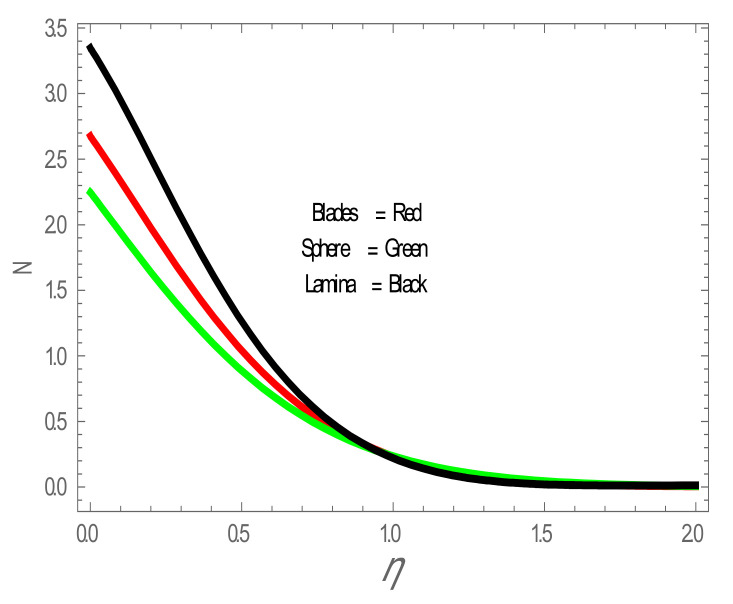
N with the effect of nanoparticles shape.

**Figure 9:**
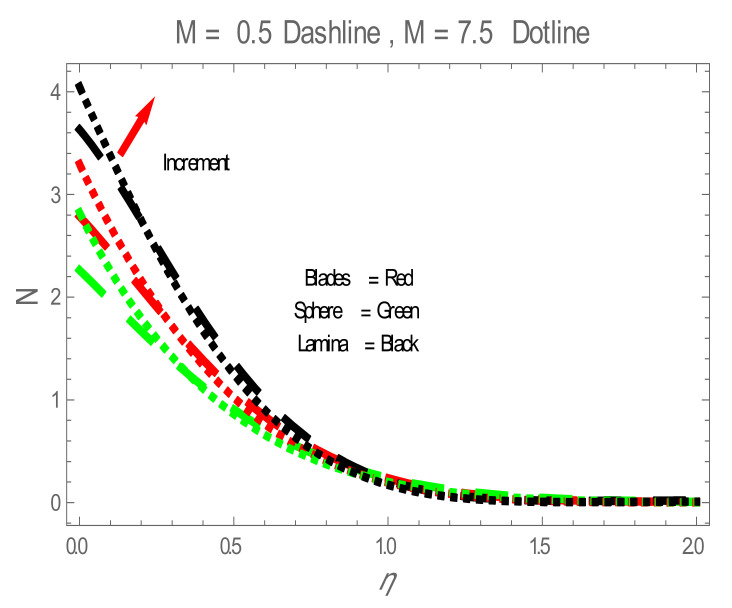
N for values of M.

**Figure 10:**
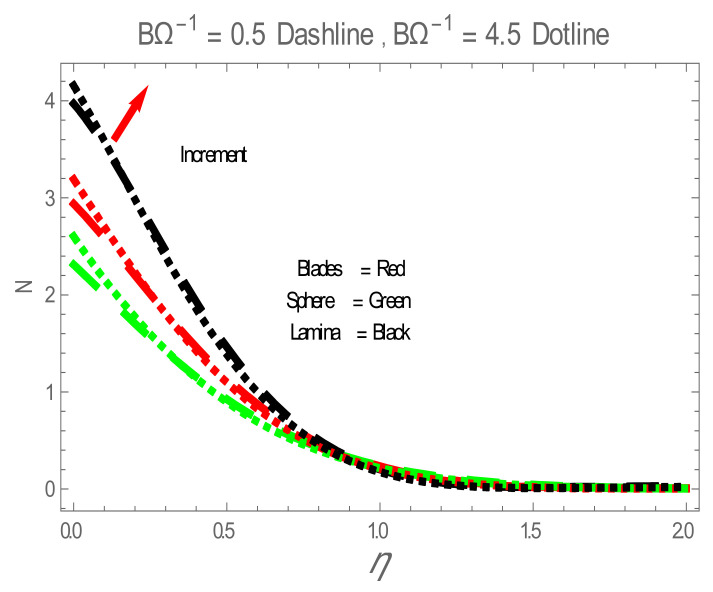
N for values of ${B\Omega }^{-1}\;$.

**Figure 11:**
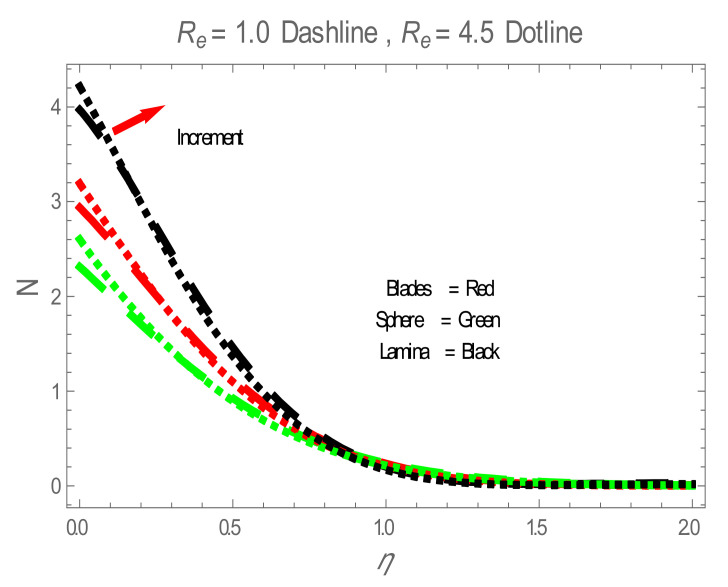
N for values of ${\;R}_{e}\;$.

**Figure 12:**
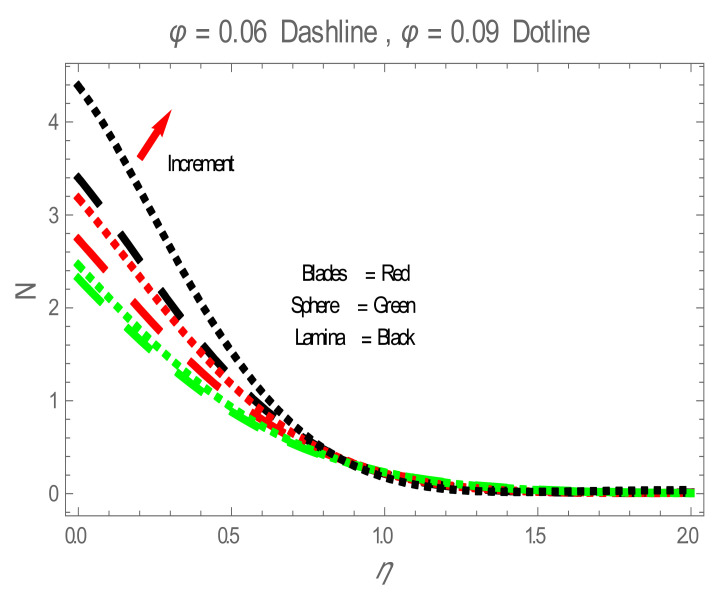
N for values of ϕ .

**Table 1:** Thermo-physical properties.

Physical Properties	Ag	Water
ρ (kg/m^3^)	10,500	997.1
Cp (J/kg K)	235	4179
*k* (W/m K)	429	0.60

**Table 2:** Shape factor (m) parameters.

Shapes	Blades	Sphere	Lamina
m	8.6	3	16.1576

**Table 3:** Results comparison for Nusselt number $\left(Nu\right)$ when M = ϕ = 0.

Nu	Present Results	Govindaraju et al. [1]	Wang Results [21]
0.7	0.0654	0.065562	0.0656
0.2	0.1690	0.169089	0.1691
70	6.4682	6.462200	6.4622

## Nomenclature

*T*	Temperature of nanofluid (k)
*u, v*	The velocity components (m/s)
Ψ	Stream function (m^2^/s)
$k_{n_{f}}$	Thermal conductivity of the nanofluid (W/m K)
$k_{f}$	Thermal conductivity of the fluid (W/m K)
$k_{s}$	Thermal conductivity of the solid (W/m K)
${\left(Cp\right)}_{nf}\;$	Specific heat capacity of the nanofluid (J/kg K)
${\left(Cp\right)}_{f}\;$	Specific heat capacity of the fluid (J/kg K)
${\left(Cp\right)}_{s}\;$	Specific heat capacity of the solid (J/kg K)
$\rho_{n_{f}}$	Density of the nanofluid (kg/m^3^)
$\rho_{f}$	Density of the fluid (kg/m^3^)
$\rho_{s}$	Density of the solid (kg/m^3^)
ϕ	Solid volume fraction
$\mu_{n_{f}}$	Dynamic viscosity of the nanofluid (Ns/m^2^)
$S_{a}$	Ratio of volumetric entropy (W/m^3^K)
$S_{b}$	Characteristic entropy generation rate
$\sigma_{n_{f}}$	Electric conductivity of nanofluid (S/m)
Ω	Dimensionless temperature difference
$B_{0}$	The imposed magnetic strength (kg/s^2^ A)
B	Brinkman number
$H_{a}$	Hartman number
Pr	Prandtl number
m	Shape variable
$n_{f}$	Nanofluid
f	Liquid
f	Solid

## Abbreviations

The following abbreviations are used in this manuscript:

MHD	Magnetohydrodynamic
PDEs	Partial differential equations
ODEs	Ordinary differential equations
HAM	Homotopy analysis method
